# “They make a difference”: a qualitative study of providers’ experiences of peer support in outpatient clinics for people living with HIV

**DOI:** 10.1186/s12913-022-08810-9

**Published:** 2022-11-21

**Authors:** Anita Øgård-Repål, Rigmor C. Berg, Vegard Skogen, Mariann Fossum

**Affiliations:** 1 Centre for Caring Research, Department of Health and Nursing Science, University of Agder, Southern Norway, Grimstad, Aust-Agder, Norway; 2 Reviews and Health Technology Assessments, Norwegian Institute of Public Health, Oslo, Norway; 3 Department of Community Medicine, University of Tromsø, The Arctic University of Norway, Tromsø, Norway; 4 Institute for Clinical Medicine, University of Tromsø, The Arctic University of Norway, Tromsø, Norway; 5 Department of Infectious Diseases, Medical Clinic, University Hospital of North Norway, Tromsø, Norway

**Keywords:** HIV, Peer support, Outpatient clinics, Social support, In-depth interviews, Thematic analysis

## Abstract

**Background:**

Although the life expectancy of people living with HIV has increased, they are still often disconnected from society through stigma and discrimination. Peer support has been found to increase social support. Given the limited research on peer support from the providers’ perspective, this study explored how peer supporters experience their roles and contributions in outpatient clinics (OPCs). Additionally, healthcare professionals’ perceptions of working with peer supporters in OPCs were examined.

**Methods:**

This qualitative study included purposively selected peer supporters (*n* = 10) and healthcare professionals (*n* = 5) from five OPCs in Norway in 2020. In-depth interviews and focus group discussions were conducted in Norwegian or English, using interview guides. Interview transcripts were analysed in NVivo 12 using reflexive and collaborative thematic analysis.

**Results:**

The results show that peer supporters experience mutual support through emotional and honest interactions. Further, the peer supporters found it essential to negotiate with the service users about their preconception of HIV, confront their views through dialogue, and replicate positive experiences by being credible role models. The participants expressed that integrating peer support in the OPCs’ usual care processes increased the prospect of equitable services. Quality of peer support and role clarity were identified as critical components. The results demonstrate that emotional and honest conversations promote support between peers and that peer supporters identify a need for a reframed understanding of HIV by modelling plausible alternative interpretations and coping experiences.

**Conclusions:**

This study contributes to knowledge on how peer support can meet the needs of people living with HIV. Incorporating people living with HIV in the co-production and distribution of healthcare services may improve the knowledge and perspectives in healthcare services. However, the skill standards of peer supporters should be addressed when implementing peer support in usual care.

**Supplementary Information:**

The online version contains supplementary material available at 10.1186/s12913-022-08810-9.

## Background

At the end of 2020, approximately 37.6 million people worldwide were living with HIV, with approximately 25.4 million undergoing antiretroviral therapy (ART) [1]. Global and national actions, particularly the availability of ART treatment, have halted and reversed the AIDS epidemic and dramatically reduced HIV incidence [2], causing HIV to be increasingly described as a chronic lifelong condition (CLLC) [1].

The life expectancy of people living with HIV (PLHIV) has approached that of the general population [3]. However, they are often burdened with coinfections and comorbidities [4], with non-communicable diseases (NCDs) and mental health disorders as some of the most prevalent comorbidities [4–6]. Unfortunately, since the beginning of the epidemic, HIV infection has been associated with social stigma and prejudice. Societal reactions indicate that HIV is one of the most stigmatised diseases in almost every culture worldwide [7–9]. Societal prejudice directed towards PLHIV can be severe, harming them in numerous ways [8, 10, 11]. Being socially stigmatised negatively affects people's psychological functioning and well-being [12], and many PLHIV become disconnected from society [8, 13]. As PLHIV are often already members of marginalised groups, such as sexual minorities and people who inject drugs, they frequently experience intersectional stigma [14, 15].

The range of challenges many PLHIV experience indicates a need for continued strengthening of established healthcare services and self-management of PLHIV. In Norway, healthcare services are organised according to the Nordic healthcare model, which is based on solidarity, focusing on universal civil rights and the protection of minorities [16]. As a national standard, outpatient clinics (OPCs) located at hospitals provide free medical follow-ups and treatment to people infected with HIV [17]. Norway has a low prevalence of HIV, with 6,778 people diagnosed with the virus by the end of 2020 [18], and seems to have achieved the first UNAIDS 90–90-90 target, with approximately 93% knowing their HIV status, 98% of people living with HIV are on treatment, and 96% being virally suppressed [19, 20]. Despite this, there is a lack of expertise about HIV within the national healthcare services. Additionally, PLHIV in Norway, who often do not disclose their diagnosis, report feeling lonely [21].

Greater involvement of users in the healthcare services may contribute to increased empowerment and a more tailored, people-centred healthcare service [22, 23]. Peer support is one way of involving service users, strengthening supportive resources in healthcare services, and increasing self-management, and it is a recognised outreach method for PLHIV [24–26]. Supported by the knowledge that individuals are socially embedded [27], social support is associated with decreased anxiety and depression, and higher resilience. Given the interrelationship between social support and health [13], social support can be a potential resilience resource when PLHIV experience stress in response to HIV-related stigma [28–30]. In particular, peer support from the larger HIV community seems crucial to PLHIV; it has been found to not only increase social support but also reduce HIV-related stigma [31, 32].

Dennis et al. defined peer support as “the giving of assistance and encouragement by an individual considered equal” ([33] p. 323). This definition is reflected in WHO’s definition of individualised peer support as “one-to-one support provided by a peer who has personal experiences of issues and challenges similar to those of another peer who would like to benefit from this experience and support” ([34] p. 1). Peer supporters (PSs) offer support and encouragement to their counterparts through meetings ranging from informal visits and sharing experiences to formal appointments focused on practical information sharing. Diverse peer support models have been applied across various health contexts [24, 35, 36]. For PLHIV, peer support grew out of the 1980s activists' reactions to combat stigma, challenge discrimination, and advocate for better treatment and care. Peer support was first organised into small groups of PLHIV supporting each other and sharing knowledge. However, since the introduction of ART, peer support has become a tailored, people-centred outreach method to provide linkage and adherence to HIV medical care and support PLHIV in taking an active role in self-management of their CLLC [4, 24].

Systematic reviews of peer support indicate that the effects of peer support vary [26, 37]. Nonetheless, findings suggest that peer support is flexible enough to be applied across healthcare contexts [27–29] and that it positively affects communities, especially in middle- and low-income countries [38]. According to a qualitative metasynthesis exploring PSs' perceptions of their role across a range of disciplines, the core of the effectiveness of peer support was found to be in equalising the provider–client power differential. It places the peer supporter in a unique situation that facilitates sharing personal experiences through reciprocal relations [35]. Unlike the numerous studies investigating the effects of peer support, only a handful of studies have examined peer support for PLHIV from the providers' perspective [39–42]. These qualitative studies highlight that PSs provide valuable practical, informational, emotional, and social support, and often model healthy behaviour. In addition, these studies emphasise that PSs feel empowered and gain self-awareness through the process [41, 43].

Given the increased valuation of peer support in care for PLHIV but limited scholarly knowledge of peer support from the providers’ perspective, this study explores how PSs experience their role and contributions in OPCs. Additionally, the study explores healthcare professionals' (HP) perceptions of working with PSs in OPCs.

## Methods

### Design

This was an exploratory qualitative study with an interpretive, reflective approach to understand peer supporters’ and healthcare professionals’ sense-making related to peer support [44, 45], and gain an in-depth understanding of the participants’ lived experiences. Individual qualitative interviews [44–46] and focus group discussions (FGDs), which allowed reflections through interaction between participants [45], were used as data collection tools.

### The advisory group

Five people from the community were invited to form an advisory group because we considered it essential to include lay community experts' perspectives and feedback throughout the research process. To optimise diversity, the advisory group consisted of two user representatives (PLHIV), one representative of a non-governmental organisation, one nurse, and one medical doctor. The nurse and the medical doctor worked at separate HIV-OPCs. The two user representatives represented the male and female genders and included an immigrant and a member of a sexual minority group. The advisory group contributed to clarifying terms, exploring research questions, developing the interview guides together with the researchers, and providing continuous input to the data analysis process.

### Study setting

Norway has four regional health authorities, with hospitals located throughout the country. The setting for the peer programme (described below) was five public OPCs situated in local hospitals in Norway, two of which were university hospitals.

#### The peer programme

A peer programme for PLHIV in Norway has existed for nearly ten years. It was user-initiated and started as a part of the standard healthcare services at one user-driven OPC serving PLHIV. In 2011, a committee of PLHIV developed targets for services based on their needs and experiences. One target was to establish peer support [47, 48]. As a result of the user-involvement process, five OPCs incorporated the peer programme as part of their healthcare services for PLHIV. Since then, in larger cities, peer support for PLHIV has been provided.

through non-governmental organisations; however, opportunities to reach people in smaller cities and towns have remained limited. The HPs at the OPCs recruit PLHIV, who receives care at their clinic, to be involved in the peer programme. The HPs approached all PLHIV connected to their clinic with information about the peer support programme. PLHIV who showed interest in the programme was invited to a meeting with the HPs for more information and a reflection about competencies required to be a PS, i.ex communication skills, personal stability, willingness and ability to sufficient knowledge about self-management, as pointed out in guidelines and recommendations [49, 50]. The HPs aimed to recruit PSs with diverse experiences and cultural background.

Through the peer programme, HPs at the five OPCs aim to provide peer support to every PLHIV enrolled at the respective OPC. Once a service user agrees to meet a PS, HPs arrange and organise the meeting. Thereby the HPs ensure to connect service users with a PS without breaking confidentiality. The HPs aimed to match the service users with suitable PSs by identifying the needs and preferences of the service users and the strengths of the PSs, as recommended in CATIE Best Practice Guidelines [49]. The HPs are responsible for providing the PSs with ongoing supervision, debriefing and support in advance and directly after the peer meetings. The supervision is one-to-one support as the PSs addressed a need to debrief emotional distress and potential challenges. The one-to-one supervison was conducted by the HPs involved in the peer programme who was trained together with the PSs. The one-to-one support allows the HPs to tailor the support to different needs of PSs. In addition, the PSs organise meetings through peer networks regularly for peer discussions,. Thereby, the content of the peer programme correspond with guidelines and recommendations related to supervision of PSs [49, 50]. Although OPCs do not employ PSs, the PSs receive a payment (72 USD per consultation) as compensation for their contribution and coverage of travel expenses.

In the current study, a PS is a person living with HIV for at least five years and being virally suppressed. The PS is receiving treatment and care at the OPCs they provide peer support and is formally trained to be a PS through a training programme developed by the OPCs. The non-peer-reviewed literature of Bloomsbury Patient Network (http://www.bloomsusers.net/), Positively UK’s National Training Programme of Peer Mentors *Project 100* (http://positivelyuk.org/project-100/), and National Standards for HIV Peer Support (http://hivpeersupport.com/) inspired PSs training programme as well as implementation in OPCs. The programme included facilitation of reflections related to role description for the PS and how the PS and HP could cooperate to guide the implementation. The training programme and implementation were conducted jointly between the HPs and PS across the included OPCs. Through this training, the PS gained and developed knowledge to provide support on a variety of issues faced by PLHIV. A PS was suggested to offer guidance grounded on values of equality and thus provide an opportunity to focus the support on the immediate here- and- now needs of the service users.

### Recruitment strategy

The HPs purposively selected a sample of PSs enrolled in the OPCs and invited them to participate in the study [51]. They approached the PSs with study information, explained both verbally and in writing. The information described the study's goals and research design. We strove for maximum variation sampling and also used snowball sampling, whereby key informants suggested other participants they believed would be valuable for increasing study insights [52–54]. Given their knowledge of the service users and PSs at their OPCs, the HPs could offer invaluable assistance in securing sample variation and suitability concerning the service users’ and PSs’ knowledge and experience of the topic. In addition, the HPs involved in peer support at the OPCs were asked to participate in both individual interviews and FGDs. PSs’ and HPs’ viewpoints were collected through 14 individual interviews, followed by two focus group discussions.

The following eligibility criteria were used for PSs: 1) enrolled in HIV clinical care at one of the OPCs, 2) aged 18 years or older, and 3) experience of being a PS at least twice by the initiative of a HP at a participating OPC (minimum two weeks before the interview). Eligible HPs had to work at one of the OPCs, collaborate with PSs, and initiate peer support meetings. Both PSs and HPs needed to be willing to sign written informed consent for participation in the study. There were no study invitation refusals; all 15 individuals who were invited, agreed to participate. We covered the participants’ travel expenses and provided light refreshments during the interviews.

#### Data construction

The first author conducted face-to-face, in-depth, semi-structured interviews and FGDs during the spring and autumn of 2020. The first author informed the participants that she is a registered nurse with previous experience of FGD interviewing and qualitative methods, but limited experience with HIV. The first author met the PSs for the first time when conducting individual interviews. She met the HPs face-to-face twice prior to the interviews to discuss recruitment and provide study information.

According to the participants' convenience, interviews and FGDs were conducted at the OPCs, except for three individual interviews with PSs. Two were conducted at a café pursuant to the PSs' request, and one was conducted digitally because of the pandemic situation related to Coronavirus Disease 2019. One of the FGDs included four PSs and one HP, and the other consisted of two PSs and two HPs.

The interviews and FGDs were audiotaped with the participants’ permission and transcribed verbatim. The first author made field notes immediately after the interviews and FGDs. Data saturation was considered after each interview transcription, reading through the transcripts, and initiating coding. After the twelfth interview, we found that additional interviews did not expand or elaborate on the existing themes [51]. The participants were asked if they wanted to read the transcripts, but all declined the offer.

The nine interviews with PSs lasted 23–102 min, with an average of 60 min, and the five interviews with HPs lasted 32–52 min, with an average of 39 min. The FGDs lasted 60–99 min, with an average of 80 min.

The interview guide was not pilot-tested but developed jointly by the authors and the advisory group. The interview guides for the PSs and HPs included 16 and 13 open-ended questions, respectively, whereas the FGDs included 14 open-ended questions. The questions concerned the participants' experiences and perceptions of PSs and HPs at the OPCs. As follow-up questions for PSs, we explored HIV status disclosure experiences, their concerns and perceptions of social stigma, and how these aspects relate to their work as PSs. Further, we explored PSs' personal experiences with social support in general and related to their HIV diagnosis.

### Analysis

We conducted a reflexive and collaborative thematic analysis with an inductive approach to identify, analyse, and report patterns in the collected data. The analysis process followed the analysis phases proposed by Braun and Clarke [55–58]. In the first phase, the four researchers became familiar with the data by repeatedly reading the transcripts. In the second phase, to develop the initial codes, the NVivo software program for qualitative data analysis was used to structure the coding of the data [59]. Two of the researchers conducted this phase following Tjora's stepwise-deductive inductive approach [48] to ensure descriptive, semantic-oriented coding. Empirical close coding reduced the potential influence of researchers' presumptions and theories as well as the volume of empirical material. Through this, empirical close codes could be shared with the advisory group without risking the participants' confidentiality [60]. In the third phase, the four researchers generated themes by sorting the codes into potential larger groups according to the shared meanings underpinning them, and then searching for sub-themes and overarching themes representing several codes. This phase was completed together with the advisory group to obtain a more nuanced understanding of the data [58]. Since contradictory data were almost non-existent, the process did not result in an expansion of the themes. The fourth phase comprised a process of reviewing and refining the themes. The researchers checked the data and the coding structure several times to determine whether the overarching themes represented the data or whether there were any missing links in the analysis. In the fifth phase, the final process of defining and naming the themes was conducted to capture each theme's essence [55, 56]. The last step, writing the report, involved providing representative, illustrative quotes from the participants to illustrate the themes, and wrapping up the analytical work. The quotes are presented verbatim, except repeated words and word fillers that were deleted to improve readability. Table 1 displays examples of the coding procedure and analysis.

**Table 1:** Examples of the coding procedure

Quotes	Empirical close coding	Group	Initial theme	Sub-theme	Theme
‘I think that's what they need, or what we all need. It’s a break. Stop being afraid, stop feeling alone, stop being the only one, just being together’ (P7)	Free from the feeling of being alone	Mutual experiences of belonging	Mutual support	Reciprocal backing between the supporter and the service user	Emotionally honest conversations promote mutual support
‘There is something about credibility, in that you live with it yourself that has a greater effect and a different effect than with healthcare professionals’ (P2)	It gives greater credibility that I have experienced it myself	Role model	The power of a good example	Credible lived experiences	Negotiation of preconceptions create reframed understandings of HIV
Not everyone gets what is being said. People have a lot of pictures and ideas in their head so that what is said is sorted into the pictures that are already there, which can be very distorted according to reality (P8)	Fear of stigma because of preconceptions	Confronting preconceptions	To challenge the individual preconception of HIV	Replicating positive experiences	Negotiation of preconceptions create reframed understandings of HIV

### Trustworthiness of the results

Several strategies were used to enhance the credibility of the results [51, 56, 61]. Data from both PSs and HPs were included, an advisory group was involved, and the first and last authors analysed the data separately and arrived at a consensus on their interpretations. The study and its findings are auditable, as we have preserved the documentation of the process for developing themes.

Furthermore, recognising that we, the researchers, are ‘outsiders’ not living with HIV, we needed to acknowledge how this could affect our situatedness in the project and the outcomes [62]. The final step of the analysis process aimed to provide a report of the perceptions and experiences of the participants deemed most salient by the researchers. Although this could constitute bias and allow the researchers to influence what is presented, cooperation with the advisory group provided an opportunity to ensure that the analysis process produced a valid and reliable report [56]. The advisory group's perspective was crucial in contextualising the data and, thereby, the trustworthiness of the data. We believe the dialogue contributed to creating broad and rich knowledge that the researchers alone could not have created, and increased the transferability of the result to other similar settings [63].

### Research ethics

The study was approved by the Regional Ethics Committee for Medical Research and the Norwegian Social Science Data Services. All participants were given oral and written information about the study. They were informed about the voluntary nature of participation and that they could withdraw from the project if and whenever they wished without any negative consequences. Written informed consent was thereafter obtained from each participant. They were required to indicate that they understood the purpose of the research and consented to participate before the interview started. Furthermore, they were informed that all data were anonymous, that their confidentiality was safeguarded, and that the data were stored following the applicable rules and guidelines for storing research material.

The manuscript preparation adhered to the 32-item checklist for interviews and focus groups, *criteria for reporting qualitative research* (COREQ) [64].

## Results

### Description of participants

We interviewed 15 individuals including 10 PSs and 5 HPs. There were nine women and six men, aged 37–65 years (mean, 49 years). All ten PSs had attended the peer support training organised by the OPCs, and all five HPs were employed at one of the participating OPCs. Supplementary characteristics of the participants are shown in Table 2, but minimal information about each participant is provided to preserve confidentiality.

**Table 2:** Description of the Study Participants (*n* = 15)

Participant ID	Gender	Service provider ID Peer supporter (PS) or Healthcare professionals (HP)	Data Individual interviews (I) and/or focus group discussions (FGD)
P1	Male	PS	I and FGD
P2	Female	PS	I and FGD
P3	Female	PS	I
P4	Male	PS	I
P5	Male	PS	I and FGD
P6	Female	PS	I and FGD
P7	Male	PS	I
P8	Female	PS	I and FGD
P9	Male	PS	I
P10	Male	PS	FGD
P11	Female	HP	I
P12	Female	HP	I and FGD
P13	Female	HP	I and FGD
P14	Female	HP	I and FGD
P15	Female	HP	I

### Themes

The qualitative analysis revealed three overarching themes: 1) how emotionally honest conversations that involve sharing experiences promote mutual support, 2) how negotiating preconceptions create reframed individual understandings of HIV, and 3) critical components for facilitating peer support in the professional OPC setting. Each theme included different aspects that were sorted into sub-themes (Table 3).

**Table 3:** Themes and sub-themes

Emotionally honest conversations promote mutual support	Negotiation of preconceptions create reframed understandings of HIV	Critical components for facilitating peer support
•Recognisable experiences and emotions •Reciprocal backing between the peer supporter and the service user	•Credible lived experiences •Replicating positive experiences	•Integration of peer support into usual care •Skill standards •Occupying the middle ground

In our presentation of the findings below, the quotes illustrating the themes are accompanied by a number, which represents the ID of the participant who contributed the quote (Table 2).

### Emotionally honest conversations promote mutual support

The results demonstrated that talking with a PS provided support to PLHIV by sharing common emotions related to experiences living with HIV. This sharing of thoughts, experiences, and honest emotions decreased feelings of being alone with the diagnosis through reciprocal backing between peers. The participants emphasised that sharing emotions had value for both parties, the PS and the service user. As different challenges arise throughout the lifespan, peers can provide mutual support when new situations occur.

#### Recognisable experiences and emotions

PSs recognised the experiences and emotions of service users. When providing support, the PSs recalled and described their own fears as well as concealing and self-quarantine behaviours to avoid being exposed as living with HIV. They also recognised loneliness, as described below.

*I think that loneliness has to do with HIV. One isolates oneself. No one is isolating you. We choose to isolate ourselves (P3).*

PSs recognised the service users’ emotions and drew on their personal experiences of what worked for them in similar situations.

*Meeting someone who has been through the same…. We are not in the same situation, you cannot compare, but you can recognise - and it is quite strange - regardless of gender, sexual orientation, ethnicity, age, it is almost like a blueprint (P7).*

When service users met HPs at the OPCs, the latter did not share personal stories. However, HPs recognised the loneliness of the service users associated with HIV as a common outcome and believed it would be useful to offer a peer meeting. The PSs’ perception was that most people newly diagnosed with HIV had a shared response involving fear and uncertainty about meeting other people. The PSs, therefore, wanted to provide support by disclosing their personal experiences. However, our findings further show that when PSs only shared positive experiences of living with HIV, service users did not believe or recognise the presented narrative, and peer support was not considered valuable.

*The peer supporter showed that she was healthy and had taken the medication for a while, and everything was well. So that's a nice value in itself. But when she signals that there was no problem, you don't get the mastery story. What worked and made it go well? One skips a few points…they do not find a deeper and mutual connection through sharing troubled emotions. So the good thing about it is that you signal hope that there does not have to be a problem and that you can fix it just fine. While it can also be a bit strange, how is it possible that there are no problems (P13).*

#### Reciprocal backing between the peer supporter and the service user

The results show that sharing lived experiences affected both PSs and service users. PSs expressed that every peer meeting of sharing their personal stories contributed to a development in their own life, while the meetings also increased their feeling of being helpful to others. Given that their unique lived experiences and ability to create emotional closeness were crucial to their role as PSs, they reported being emotionally and personally affected by the peer meetings. Through their explored perspectives, PSs wanted to contribute to the same process of discovery and improvement in service users. Thus, they strove to make the peer meeting a safe place, a kind of sanctuary. Since the service users had disclosed their diagnosis only to a few people, if any at all, the peer support meeting was, for many, a first opportunity to interact and connect with someone who thoroughly knew them and supported them. Peer meetings seemed to contribute to a sense of mutual belongingness between peers.

*I think that’s what they need, or what we all need. It's a break. Stop being afraid, stop feeling alone, stop being the only one, just be together (P7).*

The PSs expressed that different life situations actualised uncertainty of living with HIV. However, several PSs experienced that being connected to other PLHIV led to a discourse around HIV-related topics. These discussions helped when current challenges arose in their own lives, for example, several mentioned the Coronavirus Disease 2019 pandemic as a situation that raised the need to talk to peers. HPs agreed that the necessity of meeting a peer could occur when living with a CLLC.

*We all experience fluctuations through life, some good days and some bad days. But it's just like when you have a chronic illness, and you have HIV on the top; it's just like it weighs you down a little extra in the periods where it goes down, and it's difficult. So, you may have coped living with HIV for many years, but then comes the downturn and then maybe fear from the past comes up... (P13).*

### Negotiation of preconceptions create reframed understandings of HIV

PSs found it crucial to negotiate with the service users about their preconception of HIV and replicate positive experiences by being credible role models and confronting their views through dialogue.

#### Credible lived experiences

PSs are expected to be aligned with their message by being role models in how they appear and behave in living with HIV. The information provided by PSs to the service users in peer meetings could often be the same given by the HPs. However, as the information, when provided by PSs, came through the lens of experience, it could be received as more credible by the service users.

*There is something about credibility, in that you live with it yourself that has a greater effect and a different effect than with healthcare professionals (P2).*

The PSs shared personal stories and coping strategies to increase awareness of how it is to live with HIV. The PSs believed they could normalise living with HIV as a CLLC, helping service users cope with their cognitive barriers related to HIV. The PSs perceived themselves to be living examples of “normal” people, modelling and visualising a good life, thereby contributing to a reconstruction of the unique understanding of HIV.

*It's all about normalisation. Knowing that there are others and that it's going to be fine. We are completely ordinary, and there are several of us. You are not alone. It is breaking down the barriers that society also has. Look at him; he is HIV-positive, he looks completely healthy (P1).*

#### Replicating positive experiences

PSs reported personally experiencing that meeting a peer with an alternative understanding of living with HIV as early as possible after being diagnosed helped them decrease self-stigma, negative attitudes, and shame based on their preconceptions of HIV. Consequently, based on their own positive peer meeting experiences, the PSs dared to confront and challenge opinions and fears, but in a careful and respectful manner.

According to our participants, PLHIV often lack updated, factually correct knowledge of HIV, and they interpret the information they receive through that incomplete and skewed mental frame. Moreover, they expect relatives and friends to have the same lack of updated knowledge. Thus, PLHIV fear stigmatisation and rejection when disclosing their diagnosis.

*Not everyone gets what is being said. People have a lot of pictures and ideas in their head so that what is said is sorted into the pictures that are already there, which can be very distorted according to reality (P8).*

Our participants claimed that, over time, the longer they waited, the more complex the service users found it to talk about their situation, which affected their HIV disclosure attitude. The HPs experienced that meeting a PS reduced the service users’ fears, thereby underscoring the need for newly diagnosed patients to meet a PS as early as possible. PSs hoped to negotiate with the service users about their skewed preconceptions of HIV and hopefully contribute to an adjusted understanding of HIV.

*I have experienced that their eyes get quite big when I say how long I have been HIV-positive. They ask, ‘and you are not sick?’ and they ask several times. And it's like that – ‘no, I'm not sick, I go here for a check-up and take my blood tests and live a normal life with my children. It's fine’. And that does not match their terrain at all. I think it's great to be allowed to be a part of telling them that it's going to go fine* (P2)*.*

PLHIV represent different backgrounds, both culturally and socially, and thereby carry diverse preconceptions of barriers related to HIV. HPs emphasised that if individuals already represent a minority group when diagnosed with HIV, HIV can increase their burden. They further shared that offering such individuals a conversation with a PS, who themselves cope with the diagnosis every day, is essential to stress the importance of confronting or adjusting established preconceptions of HIV.

### Critical components for facilitating peer support

Both HPs and PSs found it essential to integrate peer support services into usual care, such that every person living with HIV has the same, equal opportunity to participate in peer support in a familiar and safe environment. All participants also emphasised that it was critical to ensure specific peer support skills when providing peer support at the OPCs. Nevertheless, the PSs experienced a challenge in being “in-between” regarding providing what they believed the service users needed and attending to the HPs’ expectations.

#### Integration of peer support into usual care

Our findings revealed several reasons for integrating peer support as part of usual care at OPCs. First, the HPs at the OPCs recognised that PLHIV needed a place to meet peers. The OPCs ensure equal opportunities when delivering peer support as an integrated part of the usual care reaching out to every PLHIV in their district.

Another reason for integrating peer support into OPCs is the powerful response they received from service users. The HPs’ experiences of offering peer support at the OPCs were overwhelmingly positive.

*I feel that it gives greater security, that they develop in a short time, those who are offered to meet a peer supporter. That they lower their shoulders a little and it becomes easier afterwards (P11).*

Although several non-governmental organisations offer peer support, our findings show that service users preferred to meet a PS connected to OPCs, to a greater degree, to ensure confidentiality. According to HPs, service users often asked an HP to join the first meeting with a PS or be available after their encounter with a PS. Both PSs and HPs believed that this indicated trust in the system that the service users knew and were comfortable with. The HPs emphasised that meeting a PS should be voluntary. At the same time, they had a lifespan perspective and stressed the importance of providing peer support to every PLHIV as usual care. They also stressed that new challenges may arise in PLHIVs’ lives, which may actualise the need for peer support.

The HPs' narratives show that they integrated PSs' contributions and perspectives as part of the knowledge production at the OPCs, thereby improving the quality of healthcare services. This shows how PSs sometimes have a “bridging function,” being both a service provider and a service user. Thus, they gave HPs continuous insight into how it is to live with HIV and their perspectives on the quality of services at the OPCs.

*I have learned a lot. I have become a better, at least more conscious nurse because I dare to ask more questions than I did before, maybe a little more in-depth questions than before because I have learned a lot from peer supporters. When we talk, it is easier to get into topics that we do not necessarily address often. So, I have become more aware of holistic care. (P12).*

Despite this positive attitude towards peer support, the narratives show that peer support is not sufficiently integrated into the OPCs. The HPs clearly expressed that organising peer support is resource-intensive, and figuring out how to manage peer support efficiently is an ongoing process.

#### Skill standards

As a consequence of having peer support located at the OPCs, the HPs felt responsible for the quality of the PSs' services, and they communicated these expectations to the PSs. Although no formal qualifications are required to be a PS, they have been trained in line with the peer programme described above. Both PSs and HPs stated that peer training is essential to ensure sufficient skill standards. Once PSs had attended peer training, the HPs were better informed about what to expect and what they offered as a part of the OPC services.

*We do not want to inflict on them [the patients] anything difficult that can make life even more difficult than it is. On the contrary, we want to give them something that can help make it easier. But we have no guarantee that it is a good peer meeting. You have no control. But otherwise, I have no qualms because it brings people many good experiences (P11).*

The PSs, in turn, expressed loyalty and support towards HPs' work, especially medical advice. They struggled when the service users were reluctant to take their medications, but the PSs tried to nudge service users to follow HPs’ advice.

*I have experienced people who say that it may help to pray. I find that difficult. Then you have to say that you can do that too. You can pray if you think it is comforting to pray to God or to angels or whoever it may be, or that you have friends who are praying for you. But do not stop taking the medicines (P2).*

To minimise the possibility of conflicting medical advice, the HPs, on their part, recruited individuals they believed were best fit to be PSs in terms of their communication skills, such as their ability to listen and regulate their emotions, and their ART adherence.

*We cannot have peer supporters who suddenly make someone stop taking medications. Then it doesn't help if they otherwise are trustworthy and steady (P14).*

#### Occupying the middle ground

The PSs' narratives showed that they found being a PS a positive but challenging experience. They wanted to be both professionals, as a part of the formal healthcare system, and laypersons, with the liberty to operate more like “friends.” The PSs experienced the same challenges expressed by service users.

*I feel that people I have met wanted to date or have sex. And that's perfectly normal: You have found HIV positives like yourself and want to get in touch. Thus, it may be that they want to have sex with that person. But I do not know what is right, because I am not a professional, I am not their doctor (P4).*

Given that PSs, in addition to getting involved with the service users' emotions, had to share and handle their own feelings, HPs could help with debriefing. The PSs appreciated and found support through being an integrated part of a formal system. They expressed a need to discuss peer meetings with HPs on personal boundaries, reactions, and medication adherence. The PSs found these discussions with HPs essential but challenging. Both PSs and HPs valued confidentiality, although PSs worried about breaking the confidentiality between themselves and the service user by sharing stories with the HPs. PSs felt that this sharing of stories could be understood as disclosing a friend's secret and exemplifying paraprofessional peer support.

*Those who say that they intend to take their own lives because they believe it's no point living with HIV then you are afraid of what the person will do. If you do not tell the healthcare professionals about this because you were told not to tell, but you think this person needs help. So, I think it's important that we share such information with healthcare professionals. The problem is that if I say I have to tell the nurse, they might shut up and stop sharing (P6).*

Additionally, the PSs expressed the need for flexibility in deciding the time and place for the meetings. At the same time, they tried to personalise the support by adjusting it to the condition and need of the service user.

*It is all about the need of the individual you are meeting. I can go on a full day with someone if I have the time and energy to do so and they need it (P7).*

Likewise, the PSs feared that having meetings at the OPCs could accidentally validate HIV stigma. Therefore, they preferred to meet the service users in informal locations. The PSs experienced that meeting outside the OPC opened up the possibility of discussing other, more personal topics.

*I think you have to challenge their comfort zone. If you have a 'closed' space to make them feel safe, you confirm their feelings. It's almost a validation; you validate that we have to hide* (P4).

Nearly all PSs believed that the emotional component of peer support suggests more personal meeting surroundings. However, they also experienced that meeting informally made it more challenging to balance the role and expectations of the formal system and the service user, thus highlighting the need for them to occupy the middle ground. Because there are not-yet-clear formalised codes of conduct for PSs, they searched for some consensus of behaviour. The HPs believed that service users were sceptical of meeting an unknown PS informally outside of the OPC. HPs expressed concern that service users, especially those living in small communities, were reluctant to disclose their diagnosis, and thought that organising peer support meetings at the OPC made the service user feel safer. Even though the OPCs organised peer support as a part of their services, nearly all PSs and HPs found that they needed further dialogue and considerations concerning how and where to arrange the peer meetings.

## Discussion

This study explored PSs’ experiences of their role and contributions in providing peer support to service users in OPCs and HPs' perceptions of working with PSs in OPCs.

PSs experience mutual support through emotional and honest interactions during support meetings. Peer support at the individual and interpersonal levels for both service user and PSs is perceived as a positive experience. The results also show that the PSs and HPs experience working together and integrating peer support into usual care at the OPCs as possibly contributing to improved services. This collaboration between PSs and HPs offers PLHIV equitable opportunities within healthcare services. However, for peer support at the OPCs to be successful, considering various critical aspects is required, such as equal services, PSs’ skill standards to ensure quality care, and how PSs balance both their roles as service providers and service users.

Our findings indicate that the uniqueness of peer support lies in the emotional and honest conversation between peers. This sharing of common personal experiences has the potential for mutual support, which has been described in several studies as a core element of peer support [24, 35]. In addition, studies have shown that expressing personal emotions through social support can increase people’s resilience to stigma [30, 63, 64]. Mutual support, as experienced through peer support, can be of particular importance in “non-disclosure communities” with less access to other PLHIV sharing their experiences. Therefore, our findings add to previous work documenting the complexities of HIV, social support, and disclosure [28, 29].

Furthermore, the helper therapy principle introduced by Riessmann [65], which focuses on what the helper receives from being in the helper role, as exemplified by the PSs in the present study, is congruent with studies emphasising that PSs feel more empowered and self-aware through helping others [41, 66]. In addition, consistent with previous findings [35, 42], reciprocal backing between peers was found to increase the participants’ sense of belongingness. Human beings have the drive to form and maintain positive interpersonal relationships in which mutual care is perceived. A sense of belonging is a crucial human motivation and desire [27]. Baumeister and Leary [27] describe the anxiety arising from imagined or expected social rejection, which can be seen in the non-disclosure behaviour of PLHIV as mentioned by the HPs and PSs in this study. Given that many PLHIV in Norway report situational loneliness despite excellent treatment adherence and linkage to care [19–21], providing the opportunity for a meeting with a peer is expected to allow them to experience belongingness to a group without the anxiety of being rejected because of HIV. This supports the role of PSs at the OPCs as a potential transition from social marginalisation to active participation.

PLHIV experience an ambient cultural devaluation due to HIV, which increases negative feelings and the possibility of self-stigma [9, 14]. Moreover, our participants remind us that HIV-related stigma varies between sociocultural contexts [15]. Recognising that the societal narratives of HIV are cultural constructions situated in history offers an understanding of the narratives as multiply negotiable [44, 67]. The PSs in our study aimed to contribute to a reframed individual understanding of HIV. Further, the PSs and HPs wished to decrease the service users' internalisation of others' negative views [12] by helping them avoid absorbing the cultural narratives of HIV. They did so by presenting a positive affirmation of credible lived experiences with alternative understandings and positive coping. Therefore, our findings resonate with studies that emphasise social support assisting individuals in cognitive restructuring after negative experiences such as discrimination [68, 69].

The literature documents peer support as a flexible approach applied to varied settings [25, 26, 37]. The PSs and HPs, through their experiences of working together at the OPCs, found it crucial to adjust the peer support to the context in which it is hosted to limit peer support barriers [36]. Our findings support that a critical component is the question of how to offer equitable services. Geographical distances challenge the opportunities to meet people with shared experiences regarding a non-disclosure diagnosis of HIV. Our participants emphasised that incorporating peer support at the OPCs increases the likelihood of providing people-centred peer support as part of the usual care if and when such a need arises for people living with a CLLC [24, 36]. The findings also identify the shortcomings of the HIV response and the opportunities to address them by involving PSs in the distribution of services. PSs find themselves in a unique but complex position alternating between the service user and service provider roles. The frequent interaction between PSs and HPs described in this study enriches HPs' perspectives, which has been identified by previous studies as a critical element [35]. Thus, cooperation between PSs and HPs seems to sharpen HPs’ delivery, adding continuous perspectives and knowledge [48, 70].

Our findings reveal that we must be careful when focusing on PSs who only demonstrate the successful mastery of living with HIV, instead of sharing their vulnerability and the coping strategies they have found to be most effective in promoting new behaviours, aspects with which others can identify [36]. This can be seen as a contrast to the traditional provider–client boundaries that originated in the medical model of clinical care, where emotional attachment could be understood as professional misconduct. However, we found increased recognition of a deconstruction of power relations between PSs and HPs, where the use of self is promoted [71, 72].

The integration of PSs at the OPCs, followed by the professionalisation of the PSs' personal experiences, raises the question of who defines quality in the delivery of peer support. To acknowledge peer support without adjusting the support to the medical model and losing the core element of peer support seems challenging but essential for PSs. On the other hand, HPs have a significant responsibility for the services integrated into usual care, which is reflected in their expectation of PSs’ to maintain certain skill standards. Nevertheless, the increased recognition of modest self-disclosure among professionals contributes to HPs’ recognising using oneself in the delivery of services to increase competence [71, 72], which is prominent in our findings. However, the OPC setting for providing peer support increases the need to clarify PSs’ role, to decrease potential boundary issues [73]. The informal interactions between peers seem to provide opportunities for authentic interaction and mutuality. This authentic interaction through emotional, honest self-disclosure of shared experiences can be essential to the process [71]. Still, the use of self-disclosure demonstrates how the PSs find themselves in a unique but complex position and supports the need for peer training and emotional support for the PS as described in the peer programme to balance the different demands. This might raise the question of whether organising meetings in more informal settings supports the interaction's personal component. Therefore, the flexibility implied by PSs can be understood as a prerequisite and contrast to the peer support programme on the one hand, and raising the need for role clarity for the PSs on the other hand.

### Implications

Improved understanding of the providers' experiences related to the benefits and challenges found in this study calls for the greater availability of peer support programmes in usual care. The findings can inform the development of peer support programmes. Furthermore, an increased formalisation of the peer supporter role will benefit PSs, service users, and HPs by informing expectations. Further studies on implementing peer support in professional settings should be carried out, focusing on how HPs experience developed perspectives and care by working with PSs. Power dynamics are relevant when adding voluntarism to professional settings and imply further research. In addition, future research exploring whether peer support affects service users' perceptions of living with HIV, specifically if peer support impacts HIV-related stigma, would be valuable.

### Strengths and limitations

Few studies related to peer support and HIV from the providers’ perspective have been conducted in high-income countries, highlighting the need for further research. To the best of our knowledge, this is the first study to examine peer support for people living with HIV from the providers’ perspective in a Scandinavian country.

One strength of our study is that we explored both PSs’ and HPs' experiences, which broadened the scope of the study. In addition, participants were allowed to select the most comfortable setting to enhance the likelihood of capturing rich narrative data on sensitive topics. Moreover, the advisory group contributed an emic perspective to ensure trustworthiness, which we believe enhanced our ethical research approach. Finally, the involvement of all authors in interpreting data further strengthens the credibility of the results [51, 61].

The study also has some limitations. First, the peer support programme was at different implementation stages at the OPCs, which might have affected the participants' experiences and reflections. Second, the HPs participated in the peer support training, increasing the possibility of them being favourable in their perceptions of peer support as well as the risk that more critical voices were not included in the study. Nonetheless, the results highlight that formalising the PS’s role will benefit PSs, service users, and HPs by informing expectations and facilitating positive relationships for PSs’ time and expertise.

## Conclusion

This study contributes to existing knowledge about peer support for PLHIV and provides insights into how peer support, situated at OPCs for PLHIV, is experienced from the providers’ perspective. This study demonstrates that emotional and honest conversations promote support between peers and enhances resilience at the individual and interpersonal levels through social support. An important finding is that peer support emphasises the need for a reframed understanding of HIV by modelling plausible, alternative interpretations and positive coping experiences. Furthermore, it is essential to consider the increased knowledge of healthcare services by incorporating PLHIV into the development and distribution of services. Finally, we note that integrating peer support in OPCs’ usual care increases equalising services. However, quality of peer support and role clarity are identified as critical components and should be addressed when implementing peer support in usual care.  

## Supplementary Information

**Additional file 1.** COREQ (COnsolidated criteria for REporting Qualitative research) Checklist.

## Data Availability

The datasets used in this study are presented in this article. Approval from the NSD and the participants were only linked to this study. Further information is available from the corresponding author upon request.

## Abbreviations

ART: Antiretroviral Therapy
CLLC: Chronic Lifelong Condition
PLHIV: People Living with HIV
NCD: Non-Communicable Diseases
OPC: Out-Patient Clinics
